# Controlling for Response Biases in Self-Report Scales: Forced-Choice vs. Psychometric Modeling of Likert Items

**DOI:** 10.3389/fpsyg.2019.02309

**Published:** 2019-10-15

**Authors:** Rodrigo Schames Kreitchmann, Francisco J. Abad, Vicente Ponsoda, Maria Dolores Nieto, Daniel Morillo

**Affiliations:** ^1^Department of Social Psychology and Methodology, Faculty of Psychology, Universidad Autónoma de Madrid, Madrid, Spain; ^2^Instituto de Ingeniería del Conocimiento, Madrid, Spain

**Keywords:** graded-scale, forced-choice, ipsative, social desirability, acquiescence, personality, Big Five, item response theory

## Abstract

One important problem in the measurement of non-cognitive characteristics such as personality traits and attitudes is that it has traditionally been made through Likert scales, which are susceptible to response biases such as social desirability (SDR) and acquiescent (ACQ) responding. Given the variability of these response styles in the population, ignoring their possible effects on the scores may compromise the fairness and the validity of the assessments. Also, response-style-induced errors of measurement can affect the reliability estimates and overestimate convergent validity by correlating higher with other Likert-scale-based measures. Conversely, it can attenuate the predictive power over non-Likert-based indicators, given that the scores contain more errors. This study compares the validity of the Big Five personality scores obtained: (1) ignoring the SDR and ACQ in graded-scale items (GSQ), (2) accounting for SDR and ACQ with a compensatory IRT model, and (3) using forced-choice blocks with a multi-unidimensional pairwise preference model (MUPP) variant for dominance items. The overall results suggest that ignoring SDR and ACQ offered the worst validity evidence, with a higher correlation between personality and SDR scores. The two remaining strategies have their own advantages and disadvantages. The results from the empirical reliability and the convergent validity analysis indicate that when modeling social desirability with graded-scale items, the SDR factor apparently captures part of the variance of the Agreeableness factor. On the other hand, the correlation between the corrected GSQ-based Openness to Experience scores, and the University Access Examination grades was higher than the one with the uncorrected GSQ-based scores, and considerably higher than that using the estimates from the forced-choice data. Conversely, the criterion-related validity of the Forced Choice Questionnaire (FCQ) scores was similar to the results found in meta-analytic studies, correlating higher with Conscientiousness. Nonetheless, the FCQ-scores had considerably lower reliabilities and would demand administering more blocks. Finally, the results are discussed, and some notes are provided for the treatment of SDR and ACQ in future studies.

## Introduction

In recent years, there has been a growing interest in expanding the assessment of non-cognitive characteristics such as personality traits to the field of education given their association with academic and professional achievement (Burrus et al., 2011). Meta-analytic studies indicate that the Five-Factor Model (FFM) domains are useful to predict a wide range of performance outcomes in both work (e.g., Barrick and Mount, 1991; Judge et al., 2013) and academic settings (e.g., Poropat, 2009; Richardson et al., 2012). In these scenarios, Conscientiousness has been found to provide the highest predictive power among the FFM dimensions, with an effect size as high as that of intelligence for predicting student’s grades. Along with Emotional Stability, the predictive validity of Conscientiousness over job performance is consistent across all occupations, while the remaining FFM traits are useful for specific criteria and occupations, e.g., Extraversion predicts job performance in occupations where interactions with others are important (Barrick and Mount, 1991).

The assessment of such characteristics can be useful not only for selection purposes, but also in low-stake situations, i.e., with no direct impact on the respondent’s career or opportunities, such as educational settings, as it can be a tool to enhance performance by providing individualized training (Poropat, 2014). For example, subjects with low Conscientiousness scores are expected to have difficulties with goal setting and sustained effort (Barrick et al., 1993; Judge and Ilies, 2002), while subjects with low Emotional Stability are expected to be distracted from their goals because of anxiety and self-talk (De Raad and Schouwenburg, 1996), and may benefit from meta-cognitive training.

The measurement of such characteristics has been traditionally made through self-reports with single-statement items, where respondents are asked to indicate their level of agreement, e.g., using Likert scales. This type of assessment is susceptible to the important effects of response styles such as of social desirability (SDR) and acquiescent responding (ACQ; Paulhus, 1991). The first type refers to the tendency to respond in a manner that is consistent with that which is perceived as desirable by salient others (Kuncel and Tellegen, 2009). The second type represents the preference for the positive side of the rating scale, regardless of item content (Weijters et al., 2013). Conceptually related to the last, the opposite tendency, indiscriminant disagreement, is usually called disaquiescence.

Given the variability of these response styles in the population, ignoring their possible effects on the scores may compromise the fairness and the validity of the assessments. For instance, ACQ tends to be more pronounced in children and adolescent samples, or in samples with lower levels of education, resulting in the deterioration of the psychometric properties of questionnaires (Soto et al., 2008; Rammstedt et al., 2010).

Response-style-induced errors of measurement distort the inter-item correlation matrix and can consequently affect the reliability estimates, and alter the dimensionality and the factorial structure, giving rise to misfit (e.g., Navarro-González et al., 2016; Abad et al., 2018). In a low-stakes scenario ACQ is expected to have a greater effect on factor structures than SDR, since the effects of ACQ are bidirectional. That is, ACQ will increase (decrease) the correlations among same- (opposite-) valenced items, whereas SDR will always bias up the correlation between two items affected by it (Navarro-González et al., 2016).

Regarding the effects on the relationships with external variables, both response styles, ACQ and SDR, can attenuate the predictive power over non-Likert-based criterions, given that the scores contain greater errors. Conversely, since individual differences in ACQ generalize across measures (Danner et al., 2015), it may overestimate (or underestimate) convergent validity with other external Likert-scale-based measures (Soto and John, 2019). For instance, overestimation of a positive association will occur when the trait and the criterion scales are unbalanced in the same direction i.e., when the number of positively keyed items is greater than the number of negatively keyed items for both the scales. Regarding SDR, it can also contribute to a higher association with other constructs, since it may itself be considered a meaningful personality trait (e.g., Vigil-Colet et al., 2012).

Several model-based approaches have been proposed to tackle the effect of these response styles. For example, Ferrando et al. (2009) introduced SDR and ACQ latent traits in the measurement model of Likert-scale items in the exploratory factor analysis framework. For modeling ACQ, this approach adopts the weak assumption of balance, i.e., for a chosen balanced subtest, the average loading on the content factor is of equal magnitude for both positively and negatively keyed items. For modeling SDR an independent set of SDR marker items is required. This approach has been successfully applied, and repeatedly found that in the psychometric analyses of personality self-reports, when biases are removed, the expected factorial structure is recovered better (e.g., Navarro-González et al., 2016; Morales-Vives et al., 2017).

Another prominent model for dealing with acquiescence is the Random Intercept Item Factor Analysis (RIIFA) model, which enables us to model the systematic individual tendency to respond upward or downward in the response scale (Maydeu-Olivares and Coffman, 2006). The RIIFA approach is very easy to implement, since only requires adding a single parameter, i.e., the variance of the random intercept. Additionally, although it adopts the assumption of equal importance of ACQ across the items, it is surprisingly robust to the violation of this assumption (Savalei and Falk, 2014). Several authors have found that the application of RIIFA to investigate the factorial structure of personality scales leads to clearer factor structures and improves model-to-data-fit (e.g., Aichholzer, 2014; Abad et al., 2018).

Whereas modeling response style seems an attractive solution, this type of correction can be controversial; especially for social desirability whose control does not improve criterion validity in real-life employment settings (Hough and Oswald, 2008). One reason is that SDR may relate to the content factor, and the correction might be counterproductive. Additionally, some authors have called into question the construct validity of social desirability scales (e.g., Griffith and Peterson, 2008). However, Ferrando et al. (2009) suggest that their model overrides the limitations of previous partialing or correcting approaches, and Anguiano-Carrasco et al. (2013) found that control by SDR might ameliorate faking effects.

An alternative approach to reducing response styles is to change the format of the self-assessment. Burrus et al. (2011) suggest that Forced Choice Questionnaires (FCQ) could be a promising approach in an educational context for the assessment of important non-cognitive skills that might be susceptible to faking such as work ethic and teamwork. The FCQ format consists of blocks of items with similar social desirability, which respondents must fully or partially rank according to how well the items describe them. In this way, the multidimensional FCQ format has been frequently used for measuring personality because it attenuates uniform biases such as ACQ and SDR (e.g., Cheung and Chan, 2002; Salgado and Táuriz, 2014).

Despite this, FCQ has also received some criticism because it may introduce artificial dependences among the blocks, yielding ipsative or partially ipsative scale scores, and distorting reliability estimates, factorial structure, and criterion-related validity (e.g., Brown and Maydeu-Olivares, 2013). Note that this criticism has largely ignored the fact that ipsativity is a property of the scoring method and the specific task for the respondent, not of the FCQ itself (Morillo et al., 2019). Indeed, Hicks (1970) suggested that three different types of FCQ measures should be distinguished: (1) purely ipsative, (2) quasi-ipsative, and (3) normative. For instance, if individuals fully order the items of a block, and all the scales have the same number of items, and traditional classical test theory scoring is used, a purely ipsative FCQ measure will be obtained, e.g., the sum of the scores obtained over the scales will be a constant. Conversely, FCQ scores will be quasi-ipsative if individuals only partially order the items (for a more detailed description, see Salgado and Táuriz, 2014). In recent years, the validity of personality FCQ has been analyzed meta-analytically by several authors (e.g., Salgado and Táuriz, 2014; Salgado et al., 2015; Salgado, 2017), who found higher validities for quasi-ipsative than those obtained for single-stimulus questionnaires. Additionally, Item Response Theory (IRT) models have proved to be an excellent tool to overcome the FCQ ipsativity issue, since it allows the recovery of normative scores even for purely ipsative tasks (e.g., Brown and Maydeu-Olivares, 2013; Hontangas et al., 2015; Morillo et al., 2016).

Given that it is still unclear which should be the best approach for dealing with response biases in non-cognitive assessment, this study aims to compare the validity of the FFM scores obtained in an educational setting through the application of three different approaches: (1) ignoring the SDR and Acquiescence traits in Likert-format items, (2) using a within-item multidimensional model accounting for SDR and Acquiescence in Likert-format items, and (3) using the forced-choice format within the IRT framework. Convergent, divergent and criterion-related validities are analyzed for the scores under each model. Accounting for response biases either through psychometric modeling of Likert items or using forced-choice blocks should offer more valid scores, which is expected to result in higher convergent and criterion validities.

## Materials and Methods

The dataset consisted of the responses of university students to a Personality Graded-Scale Questionnaire (GSQ), a multidimensional FCQ, and self-reported grades in the University Access Examination (UAE). Both GSQ and FCQ were composed of the same sixty statements addressing the FFM dimensions and were presented in the different response formats, i.e., Likert scale and forced-choice pairs. This study was approved by the university’s Ethics Committee, all the response data were anonymized, and all participants provided their informed consent before participating. The contents of the dataset are described below.

### Instruments

#### Personality Item Pool

The sixty statements used in the two questionnaires were taken from a 700-item pool designed to address the thirty personality facets underlying the FFM dimensions included in the NEO-PI-R model (Costa and McCrae, 1992). The items in the pool have been previously applied to a 5-point Likert scale format to a total of 1531 Psychology undergraduate students at Universidad Autónoma de Madrid, using an incomplete sampling design, and were calibrated under Samejima’s Graded Response Model (GRM; Samejima, 1968). Partial reports on the calibration study can be found in Nieto et al. (2017) and Morillo et al. (2019). In the calibration study conducted by Nieto et al. (2017), the scores based on this item pool showed good convergent validities with the NEO Five-Factor Inventory-3 (McCrae and Costa, 2007), which is a brief 60-items version of the NEO Personality Inventory-3 (McCrae et al., 2005).

One hundred and ninety-five items were excluded for having either incorrect discrimination direction, non-significant discrimination parameters, or for not having proper goodness-of-fit, i.e., *p*_*S–X^2*_ < 0.05, using the polytomous generalization of Orlando and Thissen’s (2003) S-X^2^ index (Kang and Chen, 2007).

The final sixty items were selected from the remaining 505-item pool using an Estimation-of-Distribution Algorithm (Kreitchmann et al., 2017) aimed at minimizing, in the FC-format, the sum of the squared asymptotic variance of trait estimators, assuming a MUPP-2PL model (Morillo et al., 2016) in 161051 quadrature points of the FFM main domain space (11 quadrature point per dimension), weighted by their density function assuming a standardized multivariate normal distribution. There were twelve items for each FFM domain, from which ten were direct, i.e., positively keyed, and two were inverse, i.e., negatively keyed.

#### University Access Examination

The UAE is an educational aptitude test used for undergraduate admissions in Spain. Its contents cover the Spanish Language, a Foreign Language, i.e., English, French, Italian or German, the History of Spain, and Mathematics or Latin. UAE grades are reported on a scale from 0 to 10 and represent an average score in the aforementioned contents. As will be described further, the scores in the UAE were used in this study as criterion for validity investigation. The students grades were chosen as criteria for validity given that their relationship with personality was widely investigated in meta-analytical studies (e.g., Poropat, 2009; Richardson et al., 2012), that they are not expected to be affected by social desirability or acquiescence, and because they are an important outcome measure in educational psychology.

#### Graded-Scale Questionnaire

The GSQ consisted of the sixty personality items plus four additional items measuring SDR taken from the OPERAS personality questionnaire (Vigil-Colet et al., 2013) and four items to control the quality of the participants’ responses (Maniaci and Rogge, 2014). For the latter, participants were directly instructed to mark a specific category, e.g., “For this item, please mark *agree*.” The Cronbach’s α coefficients of the subscales in the questionnaire were 0.73, 0.81, 0.78 0.77, and 0.76 for Agreeableness, Conscientiousness, Emotional Stability, Extraversion and Openness to Experience, respectively. The 4-items Social Desirability subscale presented a Cronbach’s α coefficient of 0.56. The raw scores of the GSQ in the sample described in section “Participants” of this article showed good convergent validity with OPERAS scores with the same sample, with correlations ranging from 0.61 in Agreeableness to 0.71 in Conscientiousness.

#### Forced-Choice Questionnaire

The forced-choice questionnaire was composed of 30 blocks, i.e., item pairs, assembled using the previously mentioned Estimation-of-Distribution Algorithm (Kreitchmann et al., 2017), in which constraints were set to balance the number of blocks for each pair of FFM domains, i.e., there are ten possible combinations of the five FFM domains into pairs. Three blocks addressed each of the ten pairs of FFM domains, two being positive homopolar blocks, i.e., both items are positively keyed, and one heteropolar, i.e., one positively and one negatively keyed item. As in the GSQ, three control items were included, instructing participants to mark a specific response, e.g., “For this block, please mark the first response option.”

#### Participants

Six hundred and nine Psychology undergraduate students from the first and third years at the Universidad Autónoma de Madrid (83.25% female and 16.75% male; with a mean age and standard deviation of 19.91 and 2.94, respectively) answered the GS and FC questionnaires on optical mark reader-ready response sheets.

From the initial 609 participants, eighteen were excluded for either failing, or omitting at least one control item or block, and a further thirty-three students were dropped for having at least one missing response. The final 558 participants (82.80% female and 17.20% male; with a mean age and standard deviation of 19.92 and 2.99, respectively) were randomly assigned to equally sized (*N* = 279) calibration or validation samples for data analysis. Eighty-five participants from the validation sample reported their grades in the UAE, for the criterion-related validity analyses. The students that reported their grades in the UAE were 89% female and 16% male with mean age of 20.20 and standard deviation of 4.41.

### Data Analysis

All data analysis procedures were carried out using *R* software (R Core Team, 2019), and psychometric modeling was performed with *mirt* package (Chalmers, 2012).

#### Graded-Scale Response Modeling

An initial exploratory multidimensional IRT analysis including the FFM domains was conducted using the unrecoded responses of the calibration sample to explore the factorial structure of the questionnaire and remove the eventual items that deviated from the expected FFM factorial structure. An item parameter estimation was carried out with Marginal Maximum Likelihood using the EM algorithm (Dempster et al., 1977) with Quasi-Monte Carlo integration and further rotated with oblique partially specified target rotation, i.e., target matrix contained zeros for the dimensions which items were not supposed to measure. To determine the deviation from the factor structure, item congruence coefficients were computed. Note that, although our aim was to obtain a simple item structure for the FFM domains, we also had to keep the equivalence between GSQ and FCQ. That is, excluding an item with high cross-loadings in the exploratory analysis implied also having to leave out its pair in the forced-choice format. Furthermore, the FCQ had to be well balanced in terms of block count per domain pair. Therefore, the item congruence coefficient indexes were averaged for the pairs of items in each block, and the pair with the lowest value was excluded for each of the ten pairs of domains.

Later, two confirmatory multidimensional IRT models were fitted to the validation sample dataset: (1) not controlling for SDR and ACQ, and, (2) Controlling for SDR and ACQ. The first confirmatory model was specified as a compensatory multidimensional GRM, with FFM items loading in their respective FFM main domains and facets (Eq. 1), and SDR items loading exclusively in an SDR dimension.

(1) $$P_{x_{i\,j}}\,\left({\text{θ}}_{1\,i},{\text{θ}}_{2\,i}\right)=\frac{1}{1+\text{exp}\,\left[-\left(a_{1\,j}\,{\text{θ}}_{1\,i}+a_{2\,j}\,{\text{θ}}_{2\,i}+c_{r\,j}\right)\right]}$$

where *P*_*x_ij*_ denotes the probability of subject *i* choosing *x*_*ij*_ or higher in item *j*. Parameters θ_1*i*_ and θ_2*i*_ represent the *i*th subject’s trait level in the *j*th item’s main FFM domain and facet, respectively, and *c*_*rj*_ defines an intercept term. θ_1*i*_ and θ_2*i*_ are assumed to be uncorrelated.

The second model was also specified as a compensatory GRM, but also with FFM items loading in the SDR dimension and on an additional ACQ dimension (Eq. 2). Acquiescence was defined as an approximation to the RIIFA under the IRT framework by setting the GRM scale parameters associated with the random intercept to 1, and freely estimating its variance (Primi et al., 2019).

$$P_{x_{i\,j}}\,\left({\text{θ}}_{1\,i},{\text{θ}}_{2\,i},{\text{ξ}}_{i},{\text{ζ}}_{i}\right)=$$

(2) $$\,\frac{1}{1+\text{exp}\,\left[-\left(a_{1\,j}\,{\text{θ}}_{1\,i}+a_{2\,j}\,{\text{θ}}_{2\,i}+a_{3\,j}\,{\text{ξ}}_{i}+{\text{ζ}}_{\text{i}}+c_{r\,j}\right)\right]}$$

where: ξ_*i*_ and ζ_*i*_ denote the *i*th subject’s true level of SDR and ACQ, respectively, i.e., the random intercept is ζ_*i*_ + *c*_*rj*_). Aiming at anchoring the social desirability construct from the four SDR items, the parameters for these items were estimated separately with a unidimensional GRM and later the discrimination parameters obtained were set fixed in the estimation of the full questionnaire. Furthermore, in order to have the model identified, the SDR items were assumed to load on ACQ but not to load on the FFM traits, and ACQ and SDR were assumed to be uncorrelated with the remaining latent factors (as in the model of Ferrando et al., 2009). In both confirmatory models, the correlations between FFM domains and facets were set to 0 and those between the FFM domains were freely estimated.

#### Forced-Choice Response Modeling

Recent developments in IRT modeling have overcome the ipsativity property of traditional forced-choice scoring methods and allow the estimation of normative scores. The Multi-Unidimensional Pairwise Preference (MUPP; Stark et al., 2005) model was the first proposal to do this. The MUPP understands the forced-choice response process as a result of independent evaluation on the agreement with each statement in a pair, and further decision on which to select. The probability of agreement with each statement independently is defined as a Generalized Graded Unfolding Model (GGUM; Roberts et al., 2000). Equation 3 gives the probability of endorsing one statement against the other.

(3) $$P\left(y_{i\,j}=1\right)=\frac{P\left(x_{i\,j\,1}=1\right)P\left(x_{i\,j\,2}=0\right)}{P\left(x_{i\,j\,1}=1\right)P\left(x_{i\,j\,2}=0\right)+P\left(x_{i\,j\,1}=0\right)P\left(x_{i\,j\,2}=1\right)}$$

where: *y*_*ij*_ denotes the position of the selected item on the block, i.e., 1 or 2, and *x*_*ij2*_ and *x*_*ij2*_ are the latent responses of subject *i* for items *j*_1_ and *j*_2_, respectively, being equal to 1 if respondent *i* endorses the item, and 0 if otherwise.

In this article we use a dominance variant of the MUPP model, where the probability of the agreement with each statement is given by a two-parameter logistic model (2PL): the MUPP-2PL model (Morillo et al., 2016). By replacing the GGUM by a 2PL model, the block probability function in Eq. 3 can be simplified to:

(4) $$P\left(y_{i\,j}=1|\theta_{i}\right)=\frac{1}{1+\text{exp}\,\left[-\left(a_{1\,j}\,{\text{θ}}_{i\,j\,1}-a_{2\,j}\,{\text{θ}}_{i\,j\,2}+c_{j}\right)\right]}$$

where: θ_*ij*1_ and θ_*ij*2_ denote the domains associated with items 1 and 2, respectively, in the *j*th block. By addressing both the FFM domains and facets in each statement, Eq. 5 was generalized to a within-block four-dimensional model, i.e., each block measures two domains and two facets. As in the Likert-item modeling, the correlations between FFM domains and facets were set to 0 and those between the FFM domains were freely estimated.

#### Criteria

Although the confirmatory models for GSQ and FCQ account for the FFM facets, facet scores were not included in the validity analyses, given that the reduced number of items per facet makes their scores unreliable.

The criteria for comparison between models were: (1) the reliability of the FFM domain scores, (2) the convergent and divergent validities across FFM domains and response formats, and (3) the association between FFM domain scores under each model and the students’ grades in the University Access Exam. Validity analyses used the scores of the respondents in the validation sample, calculated with the Expected-*A Posteriori* (EAP) method using the item parameter estimates from the calibration sample.

Given that traditional reliability indices, i.e., Cronbach’s α, are not applicable to forced-choice data, empirical reliability estimates from the validation sample are presented for the three models. M2-type (Maydeu-Olivares and Joe, 2006; Cai and Hansen, 2013), RMSEA and CFI fit indices were used for model evaluation in the validation sample.

## Results

### Exploratory Multidimensional IRT Analyses

Table 1 presents the standardized loadings for 60 items in the preliminary exploratory multidimensional IRT analysis. Factor congruence coefficients with the idealized structure were 0.85 for Agreeableness and Conscientiousness, 0.82 for Emotional Stability, 0.70 for Extraversion, and 0.74 for Openness to Experience. Values of factor congruence should be higher than 0.85 to be acceptable (Lorenzo-Seva and Ten Berge, 2006), indicating that either there were considerable cross-loadings or that some item loadings were low in their respective domain. Accordingly, Table 1 shows that cross-loadings were found especially for items measuring Extraversion and Emotional Stability, with considerable saturations in Emotional Stability. Model-to-data fitness for the exploratory analysis with the calibration sample were M2 = 2096.02 (*df* = 1300), RMSEA = 0.047, and CFI = 0.90, indicating a good fit.

**TABLE 1:** Standardized loadings in an exploratory IRT analysis.

**Item #**	**Paired with**	**Domain**	**Polarity**	**AG**	**CO**	**ES**	**EX**	**OE**	**C.c.**
1	36	AG	+	**0.46**	0.11	–0.03	0.02	0.00	0.97
2	26	AG	+	**0.68**	0.17	–0.12	0.22	0.05	0.91
3	52	AG	+	**0.62**	–0.01	–0.10	–0.08	0.23	0.92
4	41	AG	+	**0.46**	–0.06	0.03	0.26	0.17	0.82
5	19	AG	+	**0.48**	–0.03	0.10	0.05	–0.15	0.93
6	18	AG	+	**0.35**	0.14	0.02	0.12	0.15	0.83
7	46	AG	+	**0.68**	–0.02	0.03	0.05	0.11	0.98
8	48	AG	+	**0.35**	–0.17	0.03	0.01	0.14	0.84
9	53	AG	+	**0.51**	0.04	–0.01	–0.29	–0.15	0.84
10	27	AG	+	**0.71**	–0.03	–0.13	0.27	0.11	0.91
11	58	AG	–	**−0.57**	0.01	–0.13	0.11	0.17	0.93
12	22	AG	–	**−0.35**	0.26	0.17	0.14	**0.36**	0.58
13	25	CO	+	–0.10	**0.57**	–0.19	0.08	–0.21	0.88
14	55	CO	+	–0.25	0.22	–0.15	0.22	0.09	0.50
15	44	CO	+	0.07	**0.35**	0.02	0.22	**0.33**	0.66
16	31	CO	+	0.22	**0.34**	0.24	**−0.49**	**0.34**	0.45
17	51	CO	+	–0.14	**0.50**	0.17	0.22	0.22	0.80
18	6	CO	+	–0.03	**0.96**	–0.03	–0.01	–0.10	0.99
19	5	CO	+	0.09	**0.38**	0.03	**−0.57**	0.24	0.52
20	40	CO	+	0.15	**0.45**	0.04	0.06	0.08	0.93
21	47	CO	+	–0.04	**0.92**	–0.06	–0.02	–0.08	0.99
22	12	CO	+	0.03	**0.71**	–0.08	0.10	–0.27	0.92
23	30	CO	–	–0.02	**−0.83**	0.02	–0.09	0.11	0.99
24	56	CO	–	0.01	**−0.73**	0.24	0.01	0.25	0.90
25	13	ES	+	–0.11	–0.27	**0.78**	–0.07	–0.05	0.93
26	2	ES	+	–0.04	0.04	**0.66**	0.14	0.26	0.91
27	10	ES	+	0.06	0.29	**0.51**	0.20	0.00	0.82
28	49	ES	+	–0.08	0.11	**0.48**	0.13	**0.33**	0.78
29	45	ES	+	0.01	–0.06	0.26	**0.48**	–0.06	0.47
30	23	ES	+	–0.03	–0.25	**0.61**	0.09	–0.12	0.90
31	16	ES	+	0.25	–0.06	**0.55**	–0.21	–0.09	0.85
32	60	ES	+	–0.14	0.00	**0.64**	0.15	–0.16	0.93
33	50	ES	+	–0.05	0.06	0.18	**0.51**	0.11	0.32
34	43	ES	+	0.28	–0.10	**0.51**	–0.17	–0.10	0.82
35	37	ES	–	0.24	0.02	**−0.76**	–0.04	–0.04	0.95
36	1	ES	–	–0.09	0.05	**−0.57**	–0.28	0.05	0.88
37	35	EX	+	0.16	0.10	**0.34**	**0.49**	–0.21	0.74
38	57	EX	+	–0.14	0.23	0.06	**0.58**	0.19	0.87
39	54	EX	+	0.13	–0.16	0.01	**0.69**	–0.04	0.96
40	20	EX	+	0.12	0.14	0.24	**0.68**	–0.04	0.91
41	4	EX	+	**0.54**	0.14	0.08	**0.36**	–0.12	0.53
42	59	EX	+	0.18	–0.11	–0.02	**0.48**	–0.06	0.91
43	34	EX	+	0.03	0.05	**0.57**	0.25	–0.13	0.39
44	15	EX	+	0.12	0.14	**0.63**	0.27	0.00	0.38
45	29	EX	+	–0.01	**0.37**	0.21	**0.45**	0.20	0.69
46	7	EX	+	–0.01	0.11	0.13	0.27	–0.13	0.78
47	21	EX	–	0.04	0.05	–0.02	**−0.49**	–0.12	0.96
48	8	EX	–	0.05	0.17	0.02	**−0.59**	0.07	0.95
49	28	OE	+	0.06	–0.08	0.10	–0.17	**0.51**	0.92
50	33	OE	+	0.06	–0.10	–0.02	**0.35**	**0.46**	0.78
51	17	OE	+	0.09	0.07	–0.11	–0.12	**0.58**	0.95
52	3	OE	+	0.10	–0.05	–0.10	**0.51**	0.29	0.48
53	9	OE	+	–0.16	–0.12	0.14	–0.30	0.29	0.60
54	39	OE	+	–0.16	–0.09	–0.09	–0.09	**0.58**	0.93
55	14	OE	+	0.17	–0.04	0.02	0.21	**0.36**	0.80
56	24	OE	+	0.04	0.05	–0.01	–0.10	0.29	0.92
57	38	OE	+	–0.09	–0.04	0.01	–0.22	**0.60**	0.93
58	11	OE	+	0.12	–0.08	0.04	–0.13	**0.61**	0.95
59	42	OE	–	–0.14	0.16	–0.13	–0.19	–0.24	0.61
60	32	OE	–	–0.10	–0.03	–0.07	–0.18	–0.14	0.54

*AG = agreeableness; CO = conscientiousness; ES = emotional stability; EX = extraversion; OE = openness to experience; SDR = social desirability. Bold indicates loadings larger than 0.3 in magnitude. Shaded cells indicate items excluded for subsequent analysis. C.c. = Item congruence coefficient.*

### Confirmatory Multidimensional IRT Analyses

For the subsequent analysis, the shaded items in Table 1 were excluded using the criterion described in Eq. 1. Those items had an average congruence coefficient of 0.68, with standard deviation of 0.21. When these items were removed, Factor congruence coefficients with the idealized structure increased, varying from 0.84 (Extraversion) to 0.92 (Conscientiousness), and the items maintained had a mean congruence coefficient of 0.89, with standard deviation of 0.12 (the minimum loading of an item on its corresponding theoretical factor was 0.23). Eight items were kept for each FFM domain, from which six were direct, and two were inverse. In the FCQ, twenty blocks remained for each of the ten pairs of FFM domains. The final distribution of forced-choice blocks by item position, trait and polarity is presented in Table 2.

**TABLE 2:** Distribution of the FC blocks by item position, trait and polarity.

		**Second item**										
		**AG**		**CO**		**ES**		**EX**		**OE**		
**First item**	**Polarity**	**+**	**–**	**+**	**–**	**+**	**–**	**+**	**–**	**+**	**–**	**Total**
AG	+						1			1		2
	–									1		1
CO	+	1	1			1		1	1			5
	–					1						1
ES	+	1						1		1		3
	–							1				1
EX	+	1									1	2
	–	1										1
OE	+			1	1			1				3
	–					1						1
Total		4	1	1	1	3	1	4	1	3	1	20

*AG = agreeableness; CO = conscientiousness; ES = emotional stability; EX = extraversion; OE = openness to experience.*

The model-to-data fitness for the three confirmatory models with the validation sample is presented in Table 3. Although M2-type indices were significant in the three models, the ratios between each M2 chi-square value and its degrees of freedom (*df*) were consistently lower than 2, which can be taken as a less severe fitness criterion (Tabachnick and Fidell, 2014). The likelihood ratio test between the GSQ models (χ^2^ of change = 314.8, *df* = 41, *p* < 0.01) indicated that model with SDR and ACQ fitted the graded-scale data significantly better than that with a simple FFM structure. The RSMEA and CFI values for the three models were satisfactory, or close to acceptable.

**TABLE 3:** Model-to-data fit in the confirmatory models.

	**–2LL**	**M2**	***df***	**RMSEA**	**CFI**
GSQ with simple structure	31027.51	1312.38^∗^	758	0.051	0.88
GSQ addressing SDR and ACQ	30712.67	1001.82^∗^	717	0.038	0.94
FCQ with simple structure	6842.84	188.48^∗^	116	0.047	0.89

*SDR = social desirability; ACQ = acquiescence; GSQ = graded scale questionnaire; FCQ = forced-choice questionnaire; –2LL = –2loglikelihood; ^∗^*p* < 0.001.*

Table 4 shows that the values for the standardized factor loadings in the main FFM domains were similar between the simple structure models in both the GS and FC formats. The overall correlation between estimated standardized loadings under these models was 0.89, ranging from 0.72 in Openness to Experience, to 0.99 in Extraversion. The absolute values for the loadings in the FCQ were systematically lower than the ones for the GSQ (0.37 vs. 0.56). These results are consistent with the findings of Morillo et al. (2019) on the invariance of discrimination parameters under the GRM and the MUPP-2PL models.

**TABLE 4:** Standardized loadings in the confirmatory IRT models for graded-scale and forced-choice.

			**GSQ with**	**GSQ addressing**			**FCQ with**
			**simple structure**	**SDR and ACQ**			**simple structure**
**Item #**	**Item MD**	**Polarity**	**MD**	**MD**	**SDR**	**ACQ**	**MD**
1	AG	+	0.34	0.01	0.43	–0.05	0.39
2	AG	+	0.54	0.07	0.51	–0.04	0.59
6	AG	+	0.29	0.15	0.29	–0.04	0.14
7	AG	+	0.55	0.16	0.45	–0.04	0.39
8	AG	+	0.34	0.20	0.17	–0.05	0.14
9	AG	+	0.44	0.31	0.39	–0.04	0.32
11	AG	–	–0.61	–0.19	–0.64	–0.04	–0.37
12	AG	–	–0.23	–0.23	–0.14	–0.04	–0.28
13	CO	+	0.67	0.71	0.01	–0.03	0.28
17	CO	+	0.38	0.36	0.03	–0.05	0.46
18	CO	+	0.88	0.86	0.16	–0.01	0.61
20	CO	+	0.37	0.31	0.32	–0.04	0.16
21	CO	+	0.81	0.79	0.09	–0.02	0.61
22	CO	+	0.84	0.82	0.10	–0.03	0.06
23	CO	–	–0.81	–0.78	–0.15	–0.03	–0.63
24	CO	–	–0.83	–0.88	–0.06	–0.02	–0.58
25	ES	+	0.69	0.70	0.08	–0.03	0.57
26	ES	+	0.67	0.66	0.18	–0.03	0.39
28	ES	+	0.45	0.52	0.11	–0.04	0.25
30	ES	+	0.65	0.58	0.16	–0.04	0.59
32	ES	+	0.50	0.47	0.18	–0.04	0.39
34	ES	+	0.45	0.41	0.30	–0.04	0.09
35	ES	–	–0.66	–0.67	–0.12	–0.03	–0.59
36	ES	–	–0.56	–0.54	–0.34	–0.03	–0.47
37	EX	+	0.80	0.77	0.32	–0.03	0.44
39	EX	+	0.55	0.59	0.05	–0.03	0.31
40	EX	+	0.80	0.80	0.24	–0.03	0.55
42	EX	+	0.39	0.35	0.14	–0.05	0.14
43	EX	+	0.54	0.50	0.30	–0.04	0.42
46	EX	+	0.35	0.28	0.09	–0.05	0.11
47	EX	–	–0.39	–0.41	–0.01	–0.04	–0.43
48	EX	–	–0.49	–0.48	0.07	–0.04	–0.51
49	OE	+	0.69	0.77	–0.07	–0.03	0.39
51	OE	+	0.45	0.51	–0.03	–0.04	0.42
53	OE	+	0.41	0.40	–0.17	–0.05	0.75
54	OE	+	0.27	0.40	–0.25	–0.04	0.28
56	OE	+	0.33	0.28	0.00	–0.05	0.06
58	OE	+	0.91	0.87	0.13	–0.02	0.15
59	OE	–	–0.88	–0.88	–0.16	–0.02	–0.44
60	OE	–	–0.50	–0.68	–0.19	–0.03	–0.06
61	SDR	+	0.37		0.37	–0.05	
62	SDR	–	–0.66		–0.66	–0.04	
63	SDR	–	–0.69		–0.69	–0.04	
64	SDR	–	–0.66		–0.66	–0.04	

*AG = agreeableness; CO = conscientiousness; ES = emotional stability; EX = extraversion; OE = openness to experience; SDR = social desirability; ACQ = acquiescence; GSQ = graded scale questionnaire; FCQ = forced-choice questionnaire; MD = main domain.*

Conversely, the correlation between the loadings in the FCQ and the GSQ after accounting for SDR and ACQ was slightly lower at 0.83. Table 4 shows the values for the loadings for the Agreeableness items on the main domain were surprisingly closer to 0 after correcting for response biases, while the mean of the absolute loadings of the SDR dimension on the Agreeableness items was at least twice as high as the loadings for SDR on items from other domains (the mean of the absolute SDR loadings on items measuring Agreeableness was 0.38, while it was 0.18 or lower on the items from other domains). Similar results can be found in Navarro-González et al. (2016) where the congruence coefficient for Agreeableness drops to 0.66 after controlling for SDR, and absolute loadings for SDR on the Agreeableness items were also on average higher than those estimated on the items from the other FFM domains. These results, together with the lower empirical reliability for Agreeableness after correcting for response biases (Table 5), suggest an existing relationship between SDR and Agreeableness.

**TABLE 5:** Correlations between trait scores estimates under the three confirmatory models.

	**GSQ with simple structure**					**GSQ addressing SDR and ACQ**							**FCQ with simple structure**				
	**AG**	**CO**	**ES**	**EX**	**OE**	**AG**	**CO**	**ES**	**EX**	**OE**	**SDR**	**ACQ**	**AG**	**CO**	**ES**	**EX**	**OE**
SDR (4 items)	0.40	0.00	0.02	0.09	0.16	0.05	–0.08	–0.17	–0.08	0.06	0.81	–0.11	0.26	–0.12	0.05	0.02	–0.04
**GSQ with simple structure**																	
AG	0.63																
CO	0.02	0.92															
ES	0.01	–0.04	0.78														
EX	0.06	0.02	0.34	0.78													
OE	0.17	0.02	0.06	–0.04	0.89												
**GSQ addressing SDR and ACQ**																	
AG	0.49	–0.08	0.03	0.01	0.01	0.46											
CO	–0.04	0.97	–0.07	–0.02	–0.01	–0.09	0.83										
ES	–0.14	–0.08	0.91	0.26	0.01	–0.05	–0.09	0.70									
EX	–0.12	–0.03	0.31	0.91	–0.10	–0.07	–0.05	0.30	0.75								
OE	0.06	0.00	0.01	–0.07	0.94	–0.03	–0.02	–0.01	–0.10	0.67							
SDR	0.64	0.08	0.16	0.28	0.24	0.13	–0.01	–0.06	0.06	0.12	0.82						
ACQ	0.09	0.10	0.19	0.13	0.03	0.05	0.10	0.15	0.13	0.08	–0.04	0.55					
**FCQ with simple structure**																	
AG	0.49	–0.04	–0.03	–0.01	0.14	0.25	–0.07	–0.11	–0.11	0.08	0.37	0.00	0.49				
CO	–0.13	0.64	0.01	–0.07	0.00	–0.08	0.65	–0.01	–0.10	0.00	–0.06	0.05	–0.19	0.50			
ES	–0.01	0.05	0.66	0.36	0.06	0.00	0.02	0.64	0.34	0.02	0.18	0.11	0.03	0.06	0.66		
EX	0.14	0.03	0.16	0.51	–0.09	0.11	0.01	0.17	0.51	–0.11	0.13	0.06	0.16	–0.07	0.22	0.60	
OE	0.02	–0.13	–0.04	–0.04	0.37	0.01	–0.13	–0.01	–0.05	0.41	–0.03	–0.03	–0.03	–0.17	0.01	0.00	0.65

*Values in the diagonal of the monomethod blocks are empirical reliabilities. AG = agreeableness; CO = conscientiousness; ES = emotional stability; EX = extraversion; OE = openness to experience; SDR = social desirability; ACQ = acquiescence; GSQ = graded scale questionnaire; FCQ = forced-choice questionnaire.*

Table 5 presents the correlation coefficients between the trait EAP score estimates under each of the three confirmatory models and the SDR scores based on the four SDR items. The main diagonal represents the empirical reliabilities of the estimates. This table shows that the pattern of correlations between FFM domains was quite similar with or without modeling response biases. A moderate correlation between Emotional Stability and Extraversion can be observed with all the three models.

#### Empirical Reliabilities

The empirical reliabilities presented in the main diagonal of Table 5 were acceptable for the GSQ and somewhat lower for FCQ, which is consistent with Zhang et al. (2019). This may occur because, given their dichotomous format, pairwise forced-choice responses provide less information about the respondents’ trait levels than graded-scales. Consistent with this, previous studies (e.g., Joubert et al., 2015) have found that forced-choice blocks with more than two statements, where examinees are asked to pick the most and less representative statements, offer higher reliabilities, comparable to those from graded-scale format.

Regarding the GSQ empirical reliabilities for domain trait scores reduced after controlling response bias. This result was expected since the control implies that the common method variance is partialed out. Nevertheless, the decrease was substantially larger for Agreeableness (from 0.63 to 0.46), which might be due more to the existence of a correlation between this trait and SDR than to the social desirability of some particular items. It would imply that the SDR score obtained in the model with control of response biases may be indeed contaminated by Agreeableness variance.

#### Convergent and Divergent Validity Evidences

Table 5 also shows the correlations between ACQ and SDR with domain scores across formats. As expected from the relative proportion of positive items of each domain subtest, ACQ had a low but positive correlation with all the uncorrected FFM domain scores. These correlations were, unexpectedly, of similar size to those obtained with the corrected FFM domain scores. It might be due to the low reliability of the acquiescence measure, i.e., 0.55, and perhaps to the unbalanced nature of the scales. On the contrary, FCQ domain scores did not correlated with ACQ at all.

Regarding the SDR, we found that it correlated mainly with Agreeableness, although the size of the correlation depended on the test format. Specifically, Agreeableness estimates using FCQ responses correlated less with the 4-item-based SDR estimates than the uncorrected GSQ estimates with a simple FFM structure which after correcting for reliability attenuation: gave 0.44 and 0.60, respectively. This finding appears to suggest that the forced-choice format is somewhat more robust to social desirability than graded-scale format. The corrected GSQ-based Agreeableness estimates also had a low correlation with 4-item-based SDR estimates. However, the corrected GSQ-based Agreeableness scores had a much lower convergent validity with the FSQ -based Agreeableness scores than the uncorrected ones, i.e., the correlations corrected for reliability were 0.53 vs. 0.89.

#### Criterion Related Validity

The average of the reported grades in the UAE was 8.08, with a standard deviation of 0.91, skewness of –0.16, and kurtosis of –0.23. The criterion-related validity measures are presented in Table 6. The validities for the FCQ format are consistent with the findings of Poropat (2009) and Salgado and Táuriz (2014) in tertiary education, with a corrected correlation of around 0.20 between Conscientiousness and UAE grades, and a slightly lower correlation for Openness. On the other hand, the GSQ scores under both models illustrate moderate criterion validity evidences for Openness, but not for Conscientiousness.

**TABLE 6:** Correlations between personality scores under the three confirmatory models and the grades in the University Access Examination.

	**GSQ with**		**GSQ addressing**		**FCQ with**	
	**simple structure**		**SDR and ACQ**		**simple structure**	
	***r***	**ρ**	***r***	**ρ**	***r***	**ρ**
Agreeableness	–0.08	–0.10	–0.02	–0.03	0.02	0.03
Conscientiousness	0.01	0.01	–0.01	–0.01	0.12	0.17
Emotional Stability	–0.02	–0.02	0.04	0.05	–0.02	–0.02
Extraversion	–0.09	–0.10	–0.06	–0.07	–0.09	–0.12
Openness to Experience	0.31	0.33	0.35	0.43	0.13	0.16

*GSQ = graded scale questionnaire; forced-choice questionnaire; *r* = Pearson’s correlation coefficient; ρ = corrected correlation for reliability attenuation.*

## Discussion

The aim of this study was to investigate the effects of correcting social desirability and acquiescence biases through psychometric modeling of graded-scale items or using forced-choice format. We have found that each one of these strategies has their own advantages and disadvantages, which we suspect might depend on an interplay between the assumptions of the models, the measured traits, and the test design.

The main disadvantage of using FCQ to control response biases was its lower reliability compared with graded-scale data, even though both questionnaires have the same the number of items (i.e., 40). This lower reliability can be easily explained attending to the smaller number of measurement units (i.e., 40 items vs. 20 blocks), and to the level of measurement of these units (i.e., polytomous for GSQ vs. dichotomous for FCQ). In this sense, however, it has been observed that subjects respond significantly faster to FCQ than GSQ (Zhang et al., 2019). Therefore, reliability could be improved by increasing the number of blocks without increasing the questionnaire administration time in comparison with GSQ. Additionally, the larger GSQ reliability is in part a spurious effect that is partially due to the common method variance entered by response styles (i.e., reliabilities attenuate when they are controlled, which indicates that GSQ reliabilities are somehow inflated by them).

Regarding the correction for Acquiescence, we have found that ACQ variance was not successfully removed from GSQ-based FFM domains scores. ACQ scores did correlate slightly with GSQ-based FFM domains scores even after correcting. By contrast, and consistent with the work of Ferrando et al. (2011), the FCQ-based scores did not correlate with the ACQ, suggesting that they are more robust to this type of response bias. The adverse GSQ findings differ from the results presented by Primi et al. (2017), in which a negligible correlation (i.e., 0.03) was found between Acquiescence and the domain scores after the correction with the Random Intercept model. The greater unbalanced nature of our scales might explain our pattern of results. Although Primi et al. (2017) also used an unbalanced questionnaire in terms of polarity, the ratio between the positively and negatively keyed items in their study, i.e., 2 was lower than the one in our study which was 3. Note that in this extreme case examinees with high scores in all the traits might be less easily distinguishable from the examinees with high levels of acquiescence. This difficulty is consistent with the low reliability of the ACQ estimate. Future investigations with different polarity ratios would be useful to investigate this effect. Also, the use of more balanced GSQ scales would be desirable to improve the reliability of the ACQ estimate.

Regarding the control of social desirability, we hypothesize that each format can fail, but for different reasons. For GSQ, the results from empirical reliability and convergent validity analysis indicate that when modeling social desirability with graded-scale items, the SDR factor apparently captures part of the variance of the Agreeableness factor. This is in accordance with the results of Navarro-González et al. (2016), who found lower factor congruence for Agreeableness after correcting SDR. It suggests that Agreeableness and social desirability may be related and assuming a zero correlation in the model may be problematic. Unfortunately, the model would not be identified if this correlation was set free. Therefore, future studies may consider investigating new models allowing an estimation of the correlation between domains and SDR if necessary.

Contrary to what we expected for the convergent validity between the GSQ and FCQ scores, no substantive increase was found after correcting for response biases. The biggest improvement was for Openness to Experience scores, which was also reflected in the increment in the criterion validity with UAE grades, i.e., a corrected correlation for reliability of 0.33 without accounting for response biases, and 0.43 after accounting. Also, with regard to the criterion-related validity, an unexpected effect was observed. On the one hand, the FCQ-based Conscientiousness estimates correlated higher than the GSQ scores with the UAE criterion, being consistent with the validity evidence in meta-analysis literature (e.g., Poropat, 2009; Salgado and Táuriz, 2014). On the other hand, the correlation between the FCQ-based Openness to Experience scores, and the UAE grades were considerably lower than those using the estimates from the graded-scale data, e.g., corrected for reliability: 0.16 vs. 0.43. This effect may imply that the robustness of the FCQ blocks depends on some unknown variable. In accordance with this, in their study regarding parameter invariance between item formats, Lin and Brown (2017) suggest that the way in which items are presented may influence the psychometric qualities of the questionnaires. The authors argue that, as in the graded-scale format, the social desirability of the statements in a block may affect the decision about how to respond. Therefore, if the items in a block are not properly balanced in their social desirability, responses may tend toward the more socially desirable option.

By analyzing the differences within the blocks between the SDR loadings presented in Table 5, we discovered that the blocks with Conscientiousness items were better balanced in terms of SDR loadings, i.e., the average absolute differences between SDR loadings on the items is 0.13. Conversely, the blocks including Agreeableness and Openness were the worst balanced with an average absolute differences of SDR loadings of 0.41 and 0.33, respectively, which might explain the lower criterion validity. Accordingly, SDR loadings appear to be an important factor to take into consideration when assembling forced-choice questionnaires.

The balance of social desirability should be specially challenging for heteropolar blocks. Table 4 proves, in our case, that the SDR loadings are expected to have the same polarity as the loadings on the items’ FFM domains. Therefore, heteropolar blocks would be necessarily unbalanced with regard to SDR. Given that the inclusion of heteropolar blocks is advisable to have the MUPP-2PL properly identified (Morillo, 2018, pp. 73–104), the proposition of new models accounting for the SDR within blocks may be valuable.

Finally, we should emphasize some of the limitations of the present study. Firstly, we have only used one specific statistical model for controlling bias responses: an IRT random intercept model with SDR item markers. Therefore, we have assumed the uncorrelated nature of SDR and ACQ, or the equal importance of acquiescence for all the items. This approach is not the only one available, others exist where accounting for acquiescence can be tested (e.g., Ferrando et al., 2009; Maydeu-Olivares and Steenkamp, 2018). In the planning phase of this study, the correction of response biases was supposed to be carried out using the exploratory method of Ferrando et al. (2009). However, *ad hoc* difficulties led us to shift to a confirmatory framework. Firstly, a considerable part of the data variance was attributed to FFM facets, and it would impossible to model them jointly from an exploratory framework. Secondly, using the method published by Ferrando et al. (2009) produced results that were somewhat unstable. That is, their approach demands defining a set of equally sized positively and negatively keyed items to anchor the ACQ factor, and the results varied depending on the item assigned to this set.

An important limitation of this study was that the FCQ and the GSQ were made *ad hoc* for the current research, and some items had to be dropped in order to obtain a simple factorial structure for the confirmatory analysis. Also, their exclusion was made in way that we could maintain the equivalence of items in GSQ and FCQ in a way that neither GSQ nor FCQ were optimal. Although we decided to use cross-validation to avoid over-specifying and capitalizing on chance, we recommend that similar studies should be performed with more items.

## Data Availability Statement

The datasets generated for this study are available on request to the corresponding author.

## Ethics Statement

The studies involving human participants were reviewed and approved by Comité de Ética de la Investigación, Universidad Autónoma de Madrid. The patients/participants provided their written informed consent to participate in this study.

## Author Contributions

RK, FA, and VP proposed the original idea of the study. RK and FA carried out data analysis and contributed to analyses and the subsequent writing of this manuscript. VP, MN, and DM reviewed the manuscript and gave critical comments. All authors made substantial intellectual contributions to this study and approved the final version of the manuscript.

## Conflict of Interest

The authors declare that the research was conducted in the absence of any commercial or financial relationships that could be construed as a potential conflict of interest.
